# Bacterial genera in the fluids from apical periodontitis‐related radicular cysts: An observational study

**DOI:** 10.1111/iej.14220

**Published:** 2025-03-09

**Authors:** David Szaraz, Jan Bohm, Sabina Cerulova, Lenka Bodokyova, Zdenek Danek, Ctirad Machacek, Petra Borilova Linhartova

**Affiliations:** ^1^ Clinic of Maxillofacial Surgery University Hospital Brno Brno Czech Republic; ^2^ Clinic of Maxillofacial Surgery, Faculty of Medicine Masaryk University Brno Czech Republic; ^3^ RECETOX, Faculty of Science Masaryk University Brno Czech Republic; ^4^ Department of Pathology University Hospital Brno Brno Czech Republic; ^5^ Department of Pathology, Faculty of Medicine Masaryk University Brno Czech Republic; ^6^ Clinic of Stomatology, Faculty of Medicine Masaryk University Brno Czech Republic

**Keywords:** 16S rRNA sequencing, apical periodontitis, cystic fluid, microbiome, radicular cyst

## Abstract

**Aim:**

This study aimed to evaluate bacteriome profiles (diversity, composition and relative abundances of bacterial genera) of the fluids from apical periodontitis (AP)‐related radicular cysts (RCs).

**Methodology:**

This observational study included 29 patients with AP and RC with complete sample triplets (supragingival plaque, cryopulverized tooth and cystic fluid). The bacteriome profiles of each matrix as well as of negative controls (NCs) were examined using 16S rRNA amplicon sequencing.

**Results:**

Bacteriome profiles of cystic fluids differed from NCs in 79% of cases. The number of distinct amplicon sequence variants and Shannon index detected in cystic fluids and cryopulverized teeth were significantly lower than in paired supragingival plaque samples. Gram‐negative genera and anaerobic genera were more abundant in cystic fluids than in paired cryopulverized teeth or their supragingival plaques. The relative abundances of the genera *Prevotella_7/Prevotella*, *Fusobacterium* and *Porphyromonas* were higher in cystic fluids than in paired cryopulverized teeth and NCs; their relative abundances dominated (>20%) in individual cystic fluids. Also, DNA from the genus *Fretibacterium* was significantly more commonly found in cryopulverized teeth and cystic fluids than in supragingival plaque samples. The relative abundances of this gram‐negative bacterial genera in cryopulverized teeth differed from NCs; the difference from cystic fluids was borderline insignificant.

**Conclusions:**

Although the alpha‐diversity in the cystic fluids is much lower compared to supragingival plaques, most cystic fluids are not sterile. DNA from specific anaerobic gram‐negative bacterial genera dominated the fluids from AP‐related RCs.

## INTRODUCTION

The invasion of bacteria into the root canal system leads to apical periodontitis (AP). Besides the periapical abscess, two main possible lesions can develop in chronic AP – granuloma and radicular cyst (RC) (Braz‐Silva et al., 2019). Previous studies suggested that the development of either of these lesions depends on the periapical microbial composition (Mussano et al., 2018) and the inflammatory response (Braz‐Silva et al., 2019; Galler et al., 2021).

Multiple research methods have been developed to identify the microbial composition in the root canal system (Siqueira & Rôças, 2022) and studies have utilized various methods of tissue sampling as well as of bacteriome (i.e., the diversity and composition of bacterial DNA in the specific matrix at the time of sampling; in this paper, we will use the term ‘bacteriome’ to describe the bacterial composition at the genera level) analysis (Cerulova et al., 2023; Johnson et al., 2019).

Lately, paper point sampling or cryogrinding has become the preferred methods for periapical sampling (Hussein, 2021; Lu et al., 2022), while next‐generation sequencing (NGS) has become the gold standard for the identification of bacterial genera/strains (Bronzato et al., 2021; Manoil et al., 2020). Where cryogrinding is applied, a part of the root after apicectomy or an entire extracted tooth can be used. Precise and representative sampling is, therefore, an obvious methodological challenge (Cerulova et al., 2023; Manoil et al., 2020; Siqueira & Rôças, 2022).

Many studies employed molecular biological methods to assess the bacterial profile of infected root canals in AP (Korona‐Glowniak et al., 2021; Manoil et al., 2020; Siqueira et al., 2024). However, the literature on the analysis of the cystic fluid bacteriome is very scarce and limited to culture methods (Hrvaćanin, 2002; Iatrou et al., 1988; Scalas et al., 2013; Tek et al., 2013). Data on the bacterial diversity and composition in this field is, therefore, limited, especially where analysis with methods such as NGS, which can provide more complex results than simple culture methods, is concerned (Altaie et al., 2021; Subramanian & Mickel, 2009).

We hypothesized that the cystic fluids are not sterile and their bacteriome profiles are likely to be similar to paired cryopulverized teeth in the proportion of DNA from gram‐negative and anaerobic bacteria. At the same time, however, we expected that alpha diversities of cystic fluid bacteriomes are lower than those of supragingival plaque samples. The presented study, therefore, aimed to evaluate (using NGS) the bacteriomes of paired samples of supragingival plaques, cryopulverized teeth and cystic fluids from patients with AP and RC. We assumed that the comparison of the results from the paired samples can help us understand not only the bacteriome profile (on the genera level) of these matrices but also the dynamics of the invasion and the shifts in bacteriome composition that take place as the bacteria reach the periapex.

## METHODS

### Study design, subject selection and patient evaluation

**FIGURE 1:**
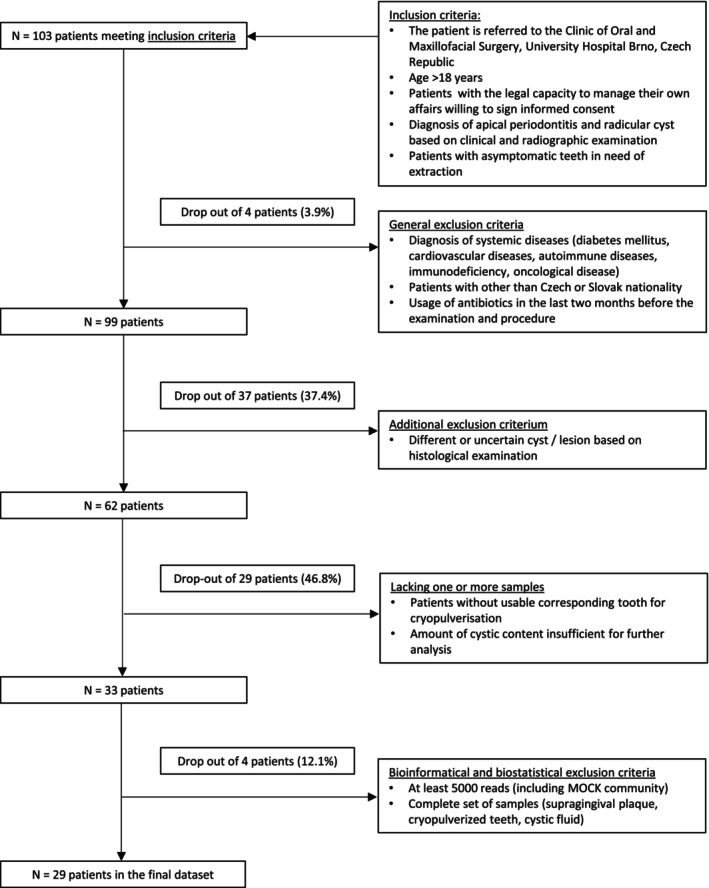
STROBE flowchart for the present study.

This explorative observational study was performed in compliance with STROBE (Strengthening the Reporting of Observational Studies in Epidemiology) guidelines. Figure 1 shows the STROBE flowchart. The research protocol was approved by the Ethics Committee of the University Hospital Brno, Czech Republic (No. 08‐120619/EK).

Patients were recruited from the pool of patients attending the Clinic of Oral and Maxillofacial surgery at the University Hospital Brno between 2020 and 2023. Only individuals >18 years of age with the legal capacity to manage their affairs who agreed to participate and signed an informed consent after being fully informed about the project, its purposes, and their role in it were included in the study. In addition, only asymptomatic teeth with primary infection in need of extraction associated with a periradicular cyst were included in the study. Patients with other than Czech or Slovak nationality, as well as those with systemic diseases (diabetes mellitus, cardiovascular diseases, autoimmune diseases, immunodeficiency, and oncological disease), pregnancy or breastfeeding, and patients taking antibiotics in the last 2 months before the examination were excluded from the study. Additional exclusion criteria were as follows: excessive crown fillings where simple extraction was not possible without crushing the crown, teeth with a missing crown, teeth with root fillings and teeth with acute gingivitis or advanced periodontitis. A complete medical history was taken from all patients, and clinical examination and panoramic X‐rays were performed. Only lesions larger than 2 cm in diameter as per X‐ray measurement were included in the study. The threshold of 2 cm was chosen to allow us to obtain enough fluid from the cyst for the analysis.

### Patient preparation, sample collection and processing, histopathological examination

Patients were requested to refrain from eating, liquid consumption and oral hygiene for at least 1 h prior to sampling. The whole sampling procedure was undertaken by an experienced surgeon. First, the supragingival plaque was sampled using a sterile swab (FLOQSwabs, COPAN, Italy) from the surface of the AP‐affected tooth indicated for extraction. Each swab was inserted into a sterile Eppendorf tube and immediately placed in a freezer at −20°C. Afterward, the patient was asked to rinse the oral cavity with a solution of 0.2% chlorhexidine for 1 min. Further, we proceeded with obtaining the cystic fluid. Taking into account the risk of contamination of the cystic fluid sample by the ubiquitous oral microbiota during the surgical removal of the cystic sac, sterile sampling of the cystic fluid was performed prior to the AP‐affected tooth extraction by puncturing the cyst with a sterile needle. Approximately 1–1.5 mL of the cystic fluid was aspirated using a 10 mL disposable sterile syringe. The fluid was then transferred into a sterile Eppendorf tube and immediately placed in a freezer at −20°C. After obtaining the cystic fluid, the corresponding AP‐affected tooth was extracted using sterile elevators and extraction forceps only. Teeth that required surgical extraction were not included in the study. Finally, the cyst was removed by elevating the mucoperiosteal flap and removing the amount of bone necessary to access the cyst, and the cyst was extirpated. The wound was sutured with resorbable suturing material. Samples of the cystic tissue were sent for histopathological evaluation, and the entire extracted AP‐affected tooth was used for the investigation of the bacteriome. During the procedure, care was taken to minimize the contamination of the surgical site with mucosa, saliva or gingiva during sampling. Extracted AP‐affected teeth were individually placed in sterile tubes and immediately placed into a freezer at −20°C.

After the procedure, the supragingival plaque samples, extracted AP‐affected teeth and cystic fluids were transported to the laboratory on ice and stored at −80°C until DNA extraction (and, in the case of extracted AP‐affected teeth, until cryogrinding). Except for the cyst tissue samples intended for histopathological confirmation, none of the samples were transported in a solution. The cystic tissue samples were placed in 4% formalin and subjected to histopathological examination at the hospital's Department of Pathology.

Each cystic tissue was sectioned on a laminar flow table and placed in a tissue embedding cassette. Cassettes were placed in an automatic tissue processor (Logos Evo, Milestone, Italy), in which the samples were automatically treated with a series of increasing ethanol concentrations, followed by xylene. The tissue was then embedded in paraffin on a tissue‐embedding unit (TES99, Thermo Fisher Scientific, USA). The paraffin blocks were serially cut into 3 μm sections for histopathological analysis. The sections were deparaffinized with xylene, hydrated in decreasing concentrations of ethanol and washed with tap water before being placed into a haematoxylin solution for 3 min. Subsequently, they were washed in tap water for 10 min, immersed in eosin for 1 min, and subsequently in water for 5 min. After that, sections were sequentially treated with 70% ethanol, absolute ethanol, acetone–xylene solution, and xylene in an automatic staining unit (Leica ST5020, Leica Biosystems, Germany). Finally, the specimen was mounted with a coverslip. Microscopic evaluation of the haematoxylin–eosin‐stained slides was performed using an optical microscope (Leica DM2000 LED, Leica Biosystems, Germany) by an experienced specialist trained in oral pathology. Cysts that were not confirmed as RCs, as well as RCs with excessive inflammation, were excluded from the study.

### Cryogrinding of the extracted AP‐affected teeth

The extracted AP‐affected teeth were individually crushed in a sterile capsule in a cryomill SPEX SamplePrep 6870 Freezer/Mill® (RMI, Czech Republic); this method is fast and suitable for homogeneous tooth cryogrinding. The capsules consist of a polycarbonate cylinder and a metallic cover and rod. First of all, it was necessary to sterilize these capsules. The polycarbonate cylinder was immersed in 0.1 M HCl for 1 h at room temperature, washed with microbial DNA‐free water and allowed to dry at laboratory temperature. Metallic parts were also washed with DNA‐free water and autoclaved. For sterility control of these capsules, 400 μL of microbial DNA‐free water was used as a capsule negative control (*N* = 6, pipetted into the capsules and then stored and treated as other samples).

After capsule sterilization, the tooth was placed into the capsule using tweezers. Cryogrinding was performed in the cryomill working on the basis of the oscillation of the metallic rod under the following conditions: pre‐cooling to liquid nitrogen temperature (−196°C) for 10 min, followed by 3 cycles of cryogrinding for 2 min with a rod frequency of 14 Hz (run‐time) separated by 2 min of cooling between cycles. The cryopulverized teeth were stored in a freezer at −80°C.

### DNA isolation and 16S rRNA amplicon sequencing analysis

The methodological approach was based on a previous review study (Cerulova et al., 2023). Microbial DNA was individually isolated from each of the three matrices (supragingival plaque, 200 mg of each cryopulverized tooth and 200 μL of each cystic fluid), from the capsule negative controls (*N* = 6, 200 μL), from negative controls (DNA‐free water, NCs, *N* = 48, 200 μL), and from a positive control (the MOCK community ZymoBIOMICS Spike‐in Control I by High Microbial Load, Zymo Research, USA) by QIAamp DNA Mini Kit (QIAGEN, Germany) following the manufacturer's instructions, using a protocol for bacterial genomic DNA isolation with the addition of lysozyme (20 mg/mL) pre‐treatment. All samples, capsule negative controls and NCs were spiked with 1000× diluted DNA from the MOCK community, which was added into the amplification reaction to avoid false negativity of the amplified DNA samples in the sequencing analysis. The samples, capsule negative controls and NCs with spiked MOCK were amplified by PCR targeting the V3/V4 regions of the gene for 16S rRNA using Illumina primers (Illumina, USA, for primer sequences see Table S1) and Q5 High‐Fidelity 2× Master Mix (New England BioLabs, USA). The PCR mix was pre‐incubated at 98°C for 30 s, which was followed by 30 cycles of a 3‐step amplification: 98°C for 10 s, 55°C for 15 s and 72°C for 30 s; then, the mixture was kept at 72°C for 30 s and cooled to 4°C. All PCR products were purified using SPRIselect (Beckman Coulter, USA). The concentration of purified samples was measured fluorimetrically using the Quant‐iT dsDNA High‐Sensitivity Assay Kit (Thermo Fisher Scientific, USA). Samples were pooled based on the concentration. Index PCR was then performed using Nextera® XT Indexes (Illumina, USA) under the following conditions: 95°C for 3 min, followed by 8 cycles of a 3‐step amplification: 95°C for 30 s, 55°C for 30 s and 72°C for 30 s; after the last cycle, the mixture was maintained at 72°C for 5 min and subsequently cooled down and kept at 4°C. All index PCR products were purified using SPRIselect (Beckman Coulter, USA). The concentration of the purified samples was measured fluorimetrically using the Quant‐iT dsDNA High‐Sensitivity Assay Kit (Thermo Fisher Scientific, USA). Samples were then pooled based on the concentration and used indexes to a final pool. The final pool was analysed by qPCR (LightCycler 480, Roche, USA), TapeStation (Agilent Technologies, USA), and fluorimetrically (Synergy™ HTX Multi‐Mode Microplate Reader, BioTek, USA). Sequencing was conducted on the MiSeq™ System (Illumina, USA) using the MiSeq Reagent Kit v3 (Illumina, USA).

### Bioinformatic analysis

We initiated our analysis by demultiplexing raw fastq files utilizing Cutadapt v.3.4 for further processing; we employed the nf‐core/ampliseq pipeline v.2.6.1. After demultiplexing, the overall quality was assessed using FastQC v.0.11.9. The reads were trimmed to a minimal Phred score of 25 and lengths of 292 bp for forward reads and 205 bp for reverse reads. Reads with mean Phred scores of less than 25 were filtered out. Reads underwent processing using the DADA2 (Divisive Amplicon Denoising Algorithm) pipeline as a part of the nf‐core/ampliseq. DADA2 1.22.0 was applied. From the merged sequences, amplicon sequence variants (ASVs) were inferred using DADA2. We used Barrnap v.0.9 for the taxonomic prediction of the kingdom of the rRNA origin to filter out Archaea, mitochondria and Eukaryotes. Taxonomic classification was performed using the DADA2 classifier (RDP naive Bayesian classifier to the SILVA 138.1 reference database). QIIME 2 v.2022.11.1 was used for the generation of absolute and relative feature/taxa count tables and the generation of basic taxa abundance plots. Additionally, alpha and beta diversity indices were computed using QIIME2. Functional profiling of sequences was conducted in Picrust2 v.2.5.0 as a part of nf‐core/ampliseq.

### Biostatistical analysis

All biostatistical analyses were conducted using the programming language R, version 4.1.2 (2021‐11‐01) (R Core Team, 2021). Tests were performed at a significance level of .05, and *p*‐values were adjusted using the Benjamini–Hochberg method within each analysis (e.g., diversity analysis, genera abundance comparison), but not across all analyses.

Taxonomic analysis utilized genus‐level data. Criteria for sample inclusion were as follows: a minimum of 5000 reads (including the spiked MOCK community).

To determine whether a sample could be considered sterile, a two‐sample Wilcoxon test with a one‐sided alternative hypothesis was performed. The test compared the Jaccard distances of the given sample to negative controls versus its distances to other non‐negative control samples. Then, *p*‐values for all samples were adjusted for multiple comparisons.

Diversity indices (number of distinct ASVs, Shannon index) were calculated using the vegan package (Oksanen et al., 2024), excluding the MOCK community. Group differences were assessed using the Wilcoxon test, applying the paired variant when applicable. In the tests of ratios, a small constant (10^−4^) was added to both the numerator and the denominator to avoid infinite values. Visualization of the data, including modified boxplots and analyses of gram‐positive/gram‐negative and aerobicity levels, was performed in the ggplot2 package (Wickham, 2016).

Principal component analysis (PCA) was conducted on centred log‐ratio (CLR) transformed genus‐level taxonomy data using the PCAtools package (Blighe & Lun, 2021). Differences in relative abundances of the 15 most abundant bacterial genera (with respect to total relative abundance across all samples) were tested using paired Wilcoxon tests.

Heatmaps of bacteriome compositions were generated using the ComplexHeatmap (Gu et al., 2016) and seriation packages (Hahsler et al., 2024). To assess the dissimilarity of samples, the Jaccard index was utilized. These heatmaps include the 25 most abundant genera, with the remainder aggregated as ‘others’. A heatmap of bacterial genera occurrences was also created, treating each genus as present in a sample if its relative abundance exceeded 0.

For the subanalysis of cystic fluids, potential contamination from other matrices was addressed by removing all ASVs found in either the supragingival plaque or the cryopulverized tooth from the cystic fluid sample. The resulting filtered ASVs were subsequently remapped to bacterial genera, following the procedures detailed in the bioinformatic analysis section.

To detect possible contaminants from chemicals used during the analysis, a series of one‐sided Wilcoxon tests was performed, comparing results from individual matrices to those from NCs. Additionally, a *z*‐test for proportions was conducted to compare the frequency of genera between results from individual matrices and NCs. For both tests, results from cryopulverized teeth and cystic fluids analyses were processed by removing ASVs identified in preceding matrices, as described above.

## RESULTS

### Selected patients, their samples and control samples

The study initially included 103 patients who met the inclusion criteria. After applying exclusion criteria, four patients (3.9%) were removed due to systemic diseases, their nationality or recent antibiotic use. Of the remaining 99 patients, 37 patients (37.4%) were excluded due to uncertain cyst or lesion diagnoses based on histological examination. Next, 29 patients (46.8% of those remaining from the previous step) were excluded for lacking a corresponding tooth for cryogrinding or an insufficient amount of cystic fluid for further analysis. Finally, four patients (12.1%) were excluded for failing bioinformatic and biostatistical criteria, such as generating fewer than 5000 reads or missing any required samples (supragingival plaque, cryopulverized tooth or cystic fluid). This process resulted in a final dataset of 29 patients (mean age 47 ± 15 years, median 49, age range 24.74 years) consisting of 19 men (65%) and nine women (35%); see the flowchart in Figure 1 for more details.

Bacteriome profiles of capsule negative controls and NCs were similar (*p* > .05); the capsule negative controls were, therefore, excluded from further analysis. The bacterial composition of all samples from patients and NCs is depicted in Figure S1. The supragingival plaque samples, cystic fluids and NCs formed distinct groups with respect to the Jaccard index, while the bacteriome compositions of cryopulverized teeth were more heterogeneous. The median proportions of reads belonging to the MOCK community were 0% for the supragingival plaque samples, 17% for the cryopulverized teeth, 3% for the cystic fluids and 63% for NCs. Overall, these results indicate that the laboratory work was well performed in maintaining the integrity of sample processing and quality control. Using the Jaccard index, bacteriome profiles of none (0%) of the supragingival plaque samples, 14 cryopulverized teeth (48%), and six cystic fluids (21%) were similar to NCs (*p*_ADJ_ < .05). According to results from NCs, DNA from genera *Flavobacterium* and *Streptococcus* were found to be the most common contaminants in the analysis; see Figure S1 and Table S2. Therefore, results for these genera should be viewed with caution.

### Paired comparison of bacteriomes in three matrices

**FIGURE 2:**
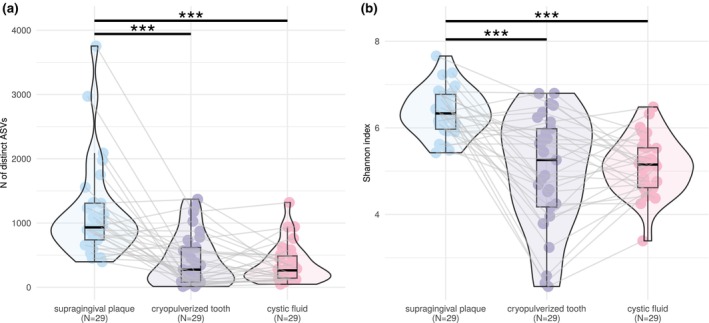
Violin boxplots displaying medians, interquartile ranges (IQR) and distributions of selected alpha‐diversity indices: number of distinct amplicon sequence variants, ASVs (a) and Shannon index (b) in paired supragingival plaque samples, cryopulverized teeth and cystic fluids. Lines connect samples from the same patient. Statistically significant differences are marked with asterisks, with : signifying *p* < .001, based on a paired Wilcoxon test with Benjamini–Hochberg adjustment.

**FIGURE 3:**
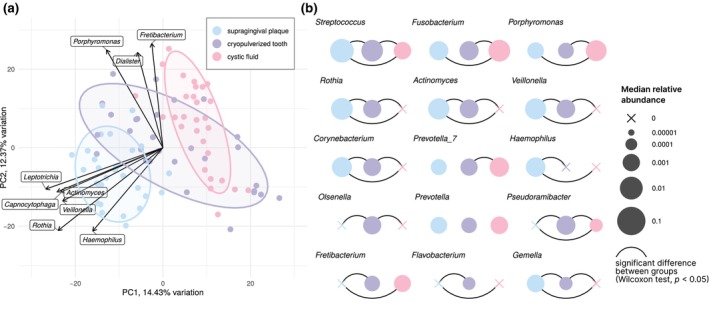
Biplot of the first and second components of principal component analysis (PCA) conducted on central log‐ratio (CLR) transformed data (a). Overview of the 15 most abundant genera (based on total relative abundance across all samples, b). Disc size represents the median value (on a log_10_ scale), and connections indicate statistically significant differences (paired Wilcoxon test, *p* < .05, adjusted within genera using the Benjamini–Hochberg method).

Both richness (measured by the number of distinct ASVs, Figure 2a) and evenness (Shannon index, Figure 2b) of supragingival plaque samples were significantly higher compared to cryopulverized teeth and cystic fluids (Wilcoxon paired test, *p*_ADJ_ < .001 for all). The PCA plot in Figure 3a demonstrates a shift in bacterial composition from the supragingival plaque to the cryopulverized tooth and, finally, to the cystic fluid. *Actinomyces, Haemophilus, Rothia and Veillonella* were among the bacterial genera associated with supragingival plaque samples, while *Porphyromonas, Fretibacterium and Dialister* were quintessential components of the bacteriome in cryopulverized teeth.

*Streptococcus* was the most abundant genus across all studied matrices; however, there were significant differences in relative abundances among them, where the decrease was observed in the direction of supragingival plaque → cryopulverized tooth → cystic fluid, see Figure 3b. *Streptococcus* dominated in supragingival plaque with relative abundances of over 20% in the majority of these samples, see Figure S1. In comparison to other matrices, the genera *Olsenella* and *Pseudoramibacter* were found in the highest relative abundances in cryopulverized teeth, see Figure 3b. The relative abundances of the genera *Fretibacterium, Fusobacterium, Porphyromonas and Pseudoramibacter* in cystic fluids were significantly higher than in paired supragingival plaque samples (Wilcoxon paired tests, *p*_ADJ_ < .05). In addition, the relative abundances of the genera *Prevotella_7*, *Fusobacterium* and *Porphyromonas* in cystic fluids were higher than in paired cryopulverized teeth (Wilcoxon paired tests, *p*_ADJ_ < .05). The results of pairwise comparisons of the relative abundances of the 15 most abundant genera are shown in Figure 3b.

**FIGURE 4:**
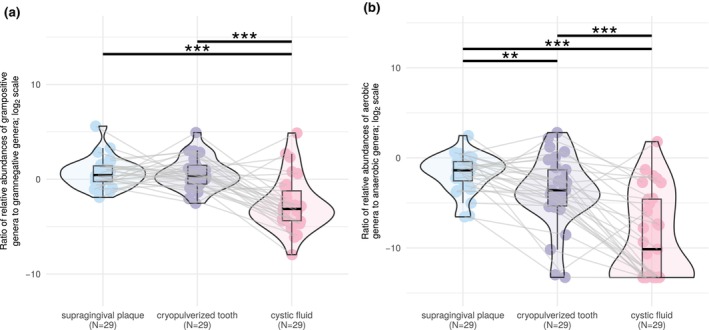
Violin boxplots displaying medians, interquartile ranges (IQR) and distributions of gram‐positive to gram‐negative ratio (a) and aerobic to anaerobic bacteria ratio (b) expressed as log_2_ fold change in paired supragingival plaque, cryopulverized teeth and cystic fluid samples. Lines connect samples from the same patient. Statistically significant differences are marked with asterisks, with ** signyfying *p* < .01 and *** *p* < .001, based on a paired Wilcoxon test with Benjamini–Hochberg adjustment.

The proportion of gram‐negative genera in cystic fluids was higher than in paired supragingival plaque samples and cryopulverized teeth (Wilcoxon paired tests, *p*_ADJ_ < .001, Figure 4a). The proportion of aerobic bacteria decreased in the direction of supragingival plaque → cryopulverized tooth → cystic fluid (Wilcoxon paired tests, *p*_ADJ_ < .01, Figure 4b).

### Bacteriome profiles of cystic fluids

**FIGURE 5:**
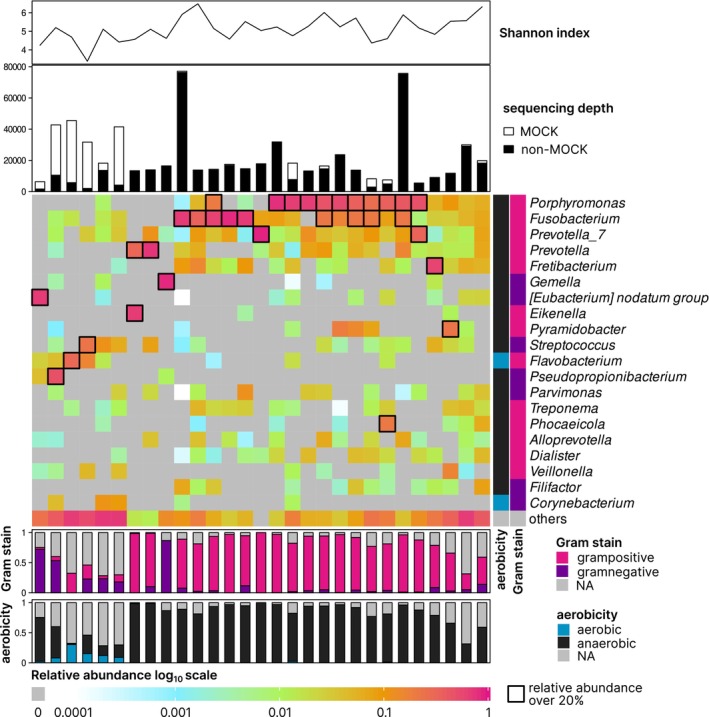
Heatmap illustrating relative abundance of 25 most abundant bacterial genera (in respect to total relative abundance across all cystic fluids) in cystic fluids. All amplicon sequencing variants (ASVs) present in paired supragingival plaque and/or cryopulverized tooth samples were removed.

In this analysis, only ASVs found exclusively in cystic fluid (but not in the paired cryopulverized tooth or supragingival plaque) were studied. The ‘cleaned’ bacteriome compositions of cystic fluids are depicted in the heatmap in Figure 5. Of the five most abundant bacterial genera in cystic fluids, high proportions (over 20%) were found for the following genera: *Porphyromonas* (ten samples), *Fusobacterium* (nine samples), *Prevotella_7*/*Prevotella* (four samples) and *Fretibacterium* (one sample). Furthermore, in the heatmap of all samples (Figure S1), the cystic fluids with high *Fusobacterium* and/or *Porphyromonas* content appeared to be mutually similar, whereas the remaining cystic fluids were scattered and were more similar to NCs.

The first occurrence of each bacterial genus in the direction of supragingival plaque → cryopulverized tooth → cystic fluid is depicted in Figure S2. There was no evidence of bacterial genera exclusive to the cystic fluids; however, *Fusobacterium*, *Porphyromonas*, *Prevotella_7* and *Prevotella* were significantly more abundant in cystic fluids than in NCs (Wilcoxon test, *p*_ADJ_ < .05), indicating that these genera are unlikely to originate from sample contamination but rather indicate their real presence in the cystic fluid. A similar trend was observed in the case of genus *Fretibacterium* in cystic fluids (*p*_ADJ_ = .059), but the relative abundance of the genus *Pseudoramibacter* did not differ from NCs in any of the matrices (Wilcoxon test, *p*_ADJ_ > .05). Detailed results for all matrices are provided in Table S2.

In addition, these results also showed that the relative abundance of the genus *Fretibacterium* in cryopulverized teeth differed from NCs (Wilcoxon test, *p*_ADJ_ < .05). The relative abundances of the genera *Olsenella* and *Pseudopropionibacterium* in this matrix differed only marginally from NCs (*p*_ADJ_ = .071 and .091, respectively).

In the supragingival plaque of AP‐affected teeth, the bacteriome profile predominantly consisted of the genera *Streptococcus*, *Actinomyces*, *Rothia*, *Veillonella*, *Leptotrichia*, *Gemella*, *Prevotella_7*, *Prevotella*, *Haemophilus* and *Selenomonas*, which were in significantly higher relative abundances than in NCs (Wilcoxon test, *p*_ADJ_ < .05). The relative abundance of the genus *Porphyromonas* in this matrix differed only marginally from NCs (*p*_ADJ_ = .097).

## DISCUSSION

AP is a polymicrobial infectious disease that stems from bacterial invasion of the root canal system. In this observational study, gram‐negative anaerobic bacterial genera *Fusobacterium*, *Porphyromonas*, *Prevotella/Prevotella_7* and *Fretibacterium* were found in fluids from AP‐related RCs. The novelty of this study lies in analysing paired samples (supragingival plaque, cryopulverized tooth, cystic fluid) from AP‐affected teeth and using the NGS technique for investigation of cystic fluid bacteriome profiles.

Two major methods are utilized for sampling the root canal bacterial content: *in vivo* (using paper points or endodontic instruments) and *ex vivo* (cryogrinding of extracted teeth or its parts). The undisputable advantage of the former is that it enables obtaining the bacterial content of the root canal exclusively in contrast to the *ex vivo* method, where significant contamination is unavoidable. On the other hand, the *in vivo* approach also has its limitations, such as missing the bacterial content of the apical ramification (which leads predominantly to obtaining planktonic bacteria but not those persisting in the biofilm or the inability to distinguish between the apical and coronal portion of the root canal). The *ex vivo* method is deemed superior when it comes to distinguishing the apical and coronal portions of the tooth when sectioning it. However, sectioning could carry a significant risk of inadvertent changes in the tooth bacteriome profile while performing it.

To prevent contamination and shifts in bacteriome composition due to manipulation with the tooth and/or heat production and cooling during sectioning, we avoided tooth sectioning prior to cryogrinding. Rather, we opted for the choice of sampling the supragingival plaque from the AP‐affected tooth, thus examining bacterial genera that are limited only to the external surface of the AP‐affected tooth referred for extraction. This gave us an idea about the bacteria colonizing both the supragingival plaque and the entire AP‐affected tooth, those limited just to the supragingival plaque and, even more importantly, those that are present in the cryopulverized tooth but not in the supragingival plaque, which indicates that these cannot originate from any surface contamination during tooth extraction.

Of studies using the *ex vivo* approach for the identification of bacterial communities in the root canal system (Alves et al., 2009; Antunes et al., 2015; Bouillaguet et al., 2018; Keskin et al., 2017; Ozok et al., 2012; Persoon et al., 2017; Qian et al., 2019; Rôças et al., 2010; Sánchez‐Sanhueza et al., 2018; Siqueira et al., 2011, 2016; Takahama et al., 2018), only three have histologically verified the periapical lesion (Antunes et al., 2015; Siqueira et al., 2016; Takahama et al., 2018). However, even two of these three studies eventually pooled the groups together (Antunes et al., 2015; Siqueira et al., 2016), not distinguishing between AP with granuloma and RC. To the best of our knowledge, our study is the first one focusing on the bacteriome of the cystic fluid of the histologically verified RCs utilizing NGS.

Cystic fluids from RCs showed a remarkably reduced bacterial alpha‐diversity, consisting mainly of anaerobic gram‐negative bacteria in line with other studies (Iatrou et al., 1988; Scalas et al., 2013; Tek et al., 2013). Our results are consistent with previously reported NGS findings (Subramanian & Mickel, 2009), where a decrease in bacterial diversity and abundance of bacterial content in the periapex compared to the root ends was observed. The interference with immunocompetent cells is one possible reason for the bacterial reduction in the periapex (Subramanian & Mickel, 2009) but the anaerobic environment due to oxygen depletion (Manoil et al., 2020) is another possible mechanism.

While DNA from typical oral bacteria, such as the genus *Streptococcus*, was usually present in all three matrices, the genus *Pseudoramibacter* was found in the cryopulverized teeth and cystic fluids but wasn't detected in the supragingival plaque samples. There are multiple possible explanations for this. One is that the bacterial genera in question might have been present in the supragingival plaque as well, however in very low, undetectable, abundances. The anaerobic environment of the cystic fluid then might have supported their growth, increasing their relative abundances and making them detectable. Another explanation could lie in the temporal factor – these bacteria might have been present in the root canal system in the past but were overgrown by other species by the time of tooth extraction (while preserved in the cystic fluid). Nevertheless, there was no difference in the relative abundances of the genus *Pseudoramibacter* in cryopulverized teeth or cystic fluids compared to NCs, which would indicate that it's a contaminant from the chemicals used during the analysis (note that both these matrices are characterized by a low abundance of bacterial genera after in silico removal of supragingival plaque bacteriome, which may lead to higher relative abundances of contaminating genera). In the case of genus *Flavobacterium*, which is a contaminant from chemicals used during the analysis (probably from the isolation kit) (Marincak Vrankova et al., 2024), only samples with a low abundance of bacterial DNA contained this genus, while supragingival plaque samples were free of this bacterial genus.

In terms of relative abundance, the populations of *Fusobacterium*, *Porphyromonas*, *Prevotella*, *Prevotella 7* and *Fretibacterium* were increasing as bacteria approached the periapex, while the contrary was true for *Streptococcus*. The genera *Fretibacterium* and (partially) *Olsenella* and *Pseudoramibacter* were specifically associated with cryopulverized teeth in our study. This confirms findings from the literature. *Fretibacterium* was previously associated with primary AP (Bouillaguet et al., 2018; Gomes et al., 2015). Besides, in a recent systematic review, *Pseudoramibacter alactolyticus*, *Olsenella uli*, *Fusobacterium* sp., *Streptococcus* sp., *Porphyromonas endodontalis* and *Prevotella* sp. were the most frequent/abundant bacterial taxa found in the apical canal of primary infections (Siqueira et al., 2024), which is perfectly consistent with our results.

*Streptococcus* sp., *Fusobacterium nucleatum and Prevotella intermedia* were previously found to be the predominant bacteria cultured from RCs (Tek et al., 2013). Our findings are in line with this observation; however, due to the NGS approach, we also found high relative abundances of other anaerobic gram‐negative bacterial genera (such as *Porphyromonas* and *Fretibacterium*) in the cystic fluids. This is not surprising, as many bacterial strains are difficult to culture, and the information obtained by a culture‐dependent method may, therefore, be incomplete. For the analysis at the lower taxonomic level, targeted molecular analysis appears to be a rational option to obtain data on clinically relevant (but difficult to culture) bacterial strains, such as *Porphyromonas* sp. and *Fretibacterium* sp. (Borilova Linhartova et al., 2020; Loesche et al., 1992; Siqueira & Rôças, 2013).

*Cutibacterium acnes* (*Propionibacterium acnes*) and *Arachnia propionica* (*Propionibacterium propionicum, Pseudopropionibacterium propionicum*) were found in the cystic fluids in previous studies (Scalas et al., 2013; Tek et al., 2013) and/or generally in the periapex (Mussano et al., 2018; Signoretti et al., 2013). The genus *Pseudopropionibacterium*, to which both bacteria previously belonged, was detected only in a few samples of cryopulverized teeth and cystic fluids but was almost entirely missing in supragingival plaques in our study. This might be caused by the fact that the genus *Pseudopropionibacterium* is more prevalent in secondary endodontic infections than in primary ones (Dioguardi et al., 2020) and as teeth with secondary infection were not included in our study, the lack of this genus in our cryopulverized tooth samples is actually not surprising.

Some limitations should be mentioned here. The number of examined subjects was lower than initially expected. The reason lies in technical problems we encountered during sample collection and evaluation. Representative samples of AP‐affected teeth suitable for cryogrinding were not available in all patients; in others, there was a lack of the cystic fluid and/or the cystic fluid could not be aspirated properly due to its consistency and density. Besides these obstacles, some specimens assumed to be AP with RC were histologically not confirmed as such or the result was ambiguous. Despite this, however, the presented study is the first one analysing bacteriome profiles of cystic fluids from RCs by the NGS technique and providing a comparison of sample triplets in patients with AP and RC (i.e., supragingival plaque, cryopulverized tooth and cystic fluid).

Another limitation pertains to teeth sectioning. Since we avoided sectioning the tooth, our study did not distinguish the bacteriome between the pulp chamber of the crown and the apical portion of the root. Nevertheless, even studies doing so provided conflicting results. Ozok et al. (2012) reported that the apical segment harboured a greater bacterial diversity than the coronal segment, while Alves et al. (2009) found similar diversity throughout the whole tooth. We have carefully considered this option but finally (considering the risks of contamination/bacterial shift associated with tooth sectioning and heating/cooling during processing), we decided that the use of a combination of paired supragingival plaques and cryopulverized AP‐affected teeth is superior for the specific identification of bacterial genera that are present predominantly in the root canal system but not (or much less) in the supragingival plaque.

Further, we only used intraoral rinsing with 0.2% chlorhexidine prior to extraction, without further external disinfection of the tooth after extraction (Qian et al., 2019). This was done in order to remove bacterial content from the external tooth biofilm and, therefore, minimize the contamination from the environment. Our results seem to confirm that this approach was quite sufficient, as the relative abundances of raw data from the cystic fluid are highly consistent with those acquired after in silico removal of ASVs from the paired supragingival plaque and cryopulverized tooth samples (see Figure S3).

The fact that we did not distinguish between the supragingival and subgingival bacteriome as we swabbed the biofilm of the crown only prior to the extraction can also be considered a limitation. However, acute gingivitis and/or advanced periodontitis were exclusion criteria and, therefore, it can be assumed that subgingival plaque would not play a major role in the bacteriome profile of the extracted AP‐affected tooth.

On the plus side, the use of NCs and the MOCK community as a positive control is a strong point of the study as it allowed the identification of potential sterile cystic fluid samples. It is also worth noting that in some analysed cryopulverized teeth, results were similar to NCs. Thanks to the methodological approach (spiking of the MOCK community into all samples), we know that this is not a result of any PCR inhibition when processing these samples.

## CONCLUSIONS

Although the alpha‐diversity in the cystic fluids is much lower compared to supragingival plaques, most cystic fluids are not sterile and are dominated by anaerobic gram‐negative bacteria. We have found high relative abundances of the genera *Porphyromonas*, *Fusobacterium*, *Prevotella_7/Prevotella and Fretibacterium* in fluids obtained from AP‐related RCs. Specific bacteria from these genera with potential clinical significance should be examined by modern approaches in order to obtain information about their quality and quantity, as well as their predisposition to sensitivity to antimicrobial therapy.

## AUTHOR CONTRIBUTIONS

David Szaraz: Writing – original draft preparation (lead), investigation (equal), data curation (supporting). Jan Bohm: Writing – original draft preparation (equal), data curation (lead), formal analysis (lead), visualization (lead). Sabina Cerulova and Lenka Bodokyova: Writing – original draft preparation (supporting), investigation (equal). Ctirad Machacek: Writing – original draft preparation (supporting), investigation (supporting). Zdenek Danek: Methodology (equal), investigation (supporting), resources (equal). Petra Borilova Linhartova: Conceptualization (lead), methodology (equal), resources (equal), writing – original draft preparation (equal), supervision (lead), project administration (lead), funding acquisition (lead). All authors revised the manuscript and approved the final version.

## FUNDING INFORMATION

The study was supported by the Ministry of Health of the Czech Republic, grant No. NU20‐08‐00205 and by a project funded by the University Hospital Brno – RVO (FNBr, 65269705).

## CONFLICT OF INTEREST STATEMENT

Authors declare no conflict of interest.

## ETHICS STATEMENT

The study was approved by the Ethics Committee of the University Hospital Brno, Czech Republic (No. 08‐120619/EK, June 12th, 2019).

## PATIENT CONSENT

The informed consent was obtained from all participants prior to their inclusion in the study and sample collection in line with the Helsinki Declaration.

## Supporting information


Figure S1

Figure S2

Figure S3

Table S1

Table S2

## Data Availability

The data for this study have been deposited in the European Nucleotide Archive (ENA) at EMBL‐EBI under accession number PRJEB84242 (https://www.ebi.ac.uk/ena/browser/view/PRJEB84242).
