# Supplementary material for: Bacterial genera in the fluids from apical periodontitis‐related radicular cysts: An observational study
Source: Int Endod J. 2025 Mar 9;58(6):902–15. doi: 10.1111/iej.14220 (PMC12065126; doi:10.1111/iej.14220)
Supplement: Supplementary file 1 — Figure S1 [file IEJ-58-902-s001.pdf]

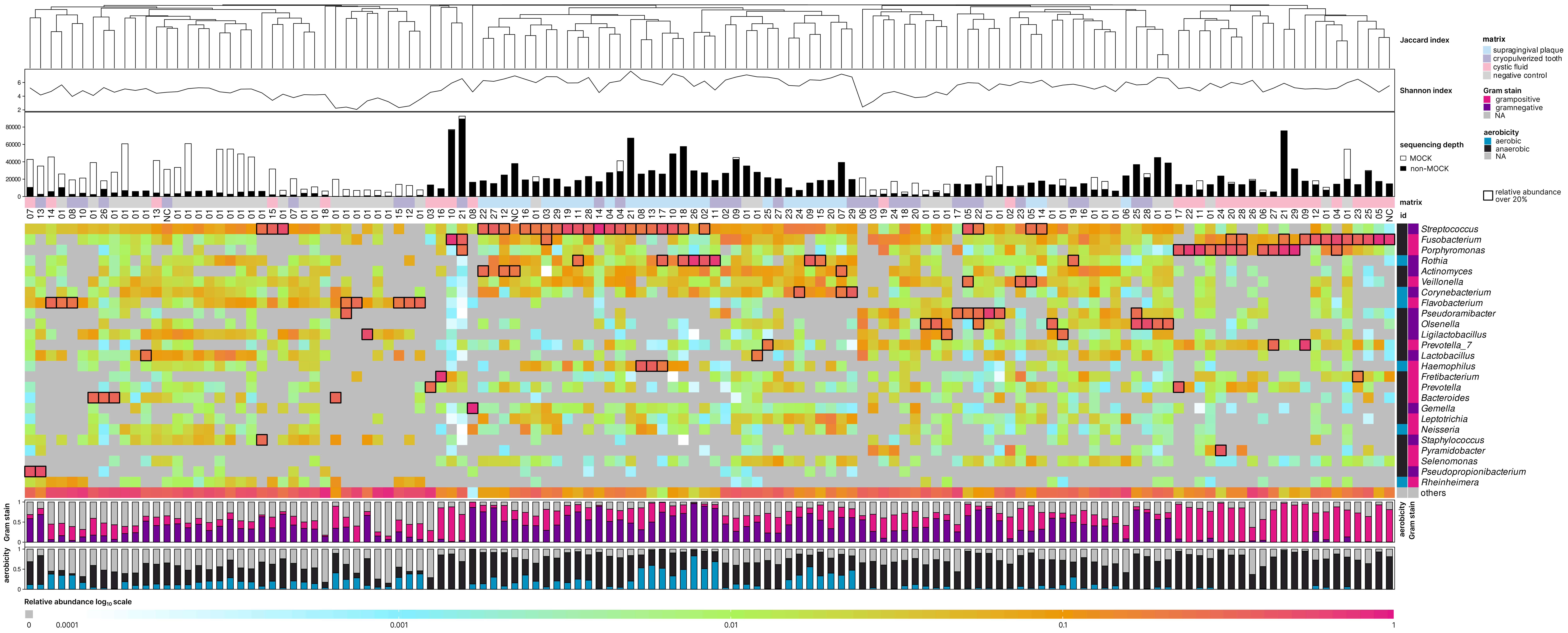


**SUPPLEMENTARY FIGURE S1.** Heatmap illustrating relative abundance of 25 most abundant bacterial genera (in respect to total relative abundance across all samples) in all samples including negative controls (NCs). Sampels are clustered based on Jaccard index. The median success of determination of Gram staining and aerobicity of bacterial genera in the samples was 73 % (the rest of the bacteriome was either not determined at the level of the genera or their Gram staining/aerobicity status is uncertain).
