# Supplementary material for: Bacterial genera in the fluids from apical periodontitis‐related radicular cysts: An observational study
Source: Int Endod J. 2025 Mar 9;58(6):902–15. doi: 10.1111/iej.14220 (PMC12065126; doi:10.1111/iej.14220)
Supplement: Supplementary file 2 — Figure S2 [file IEJ-58-902-s004.pdf]

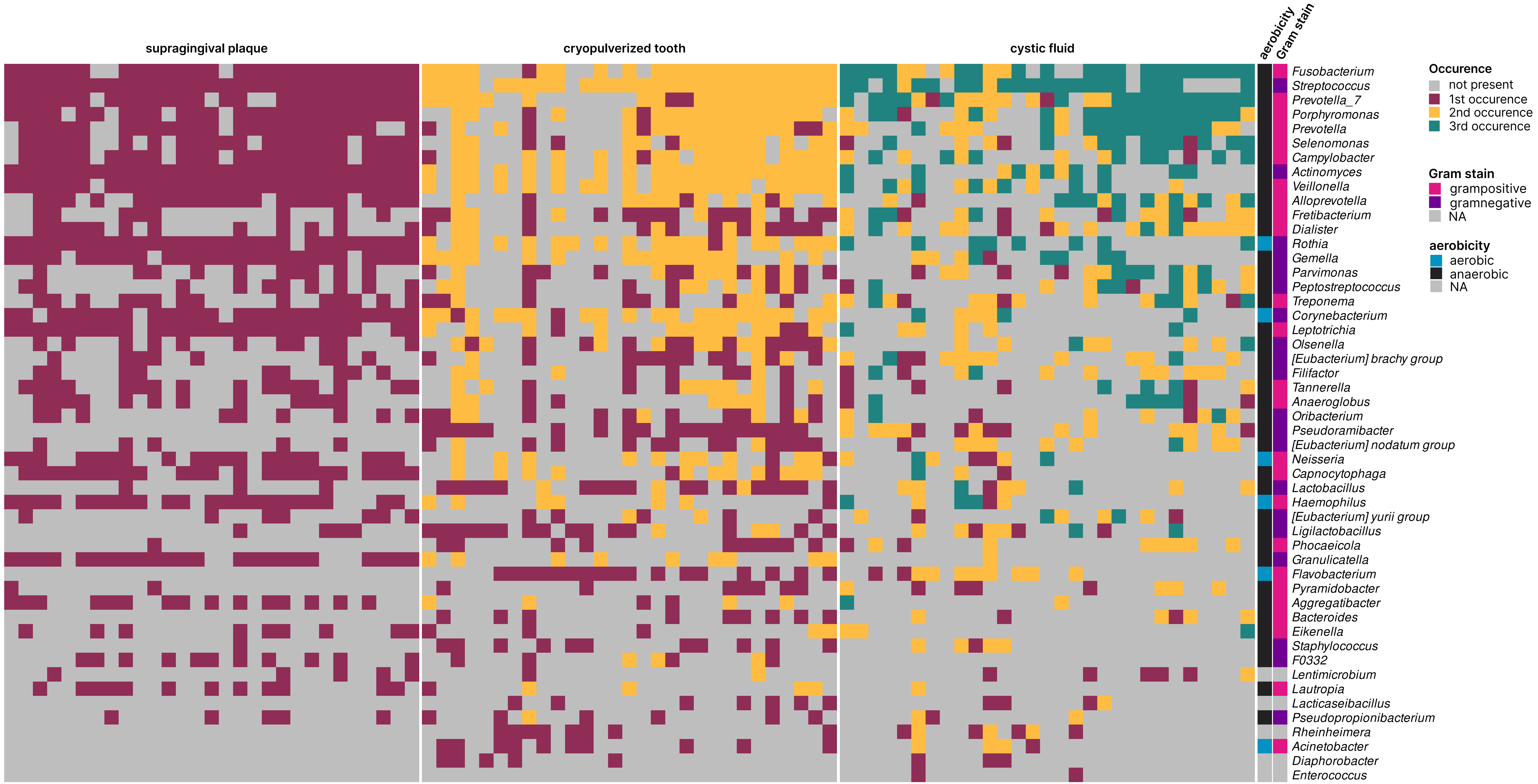

SUPPLEMENTARY FIGURE S2. An overview of fist, second and third occurrence of bacterial genera in samples from given patient in direction of supragingival plaque → cryopulveized tooth → cystic fluid.
