# Supplementary material for: Bacterial genera in the fluids from apical periodontitis‐related radicular cysts: An observational study
Source: Int Endod J. 2025 Mar 9;58(6):902–15. doi: 10.1111/iej.14220 (PMC12065126; doi:10.1111/iej.14220)
Supplement: Supplementary file 3 — Figure S3 [file IEJ-58-902-s005.pdf]

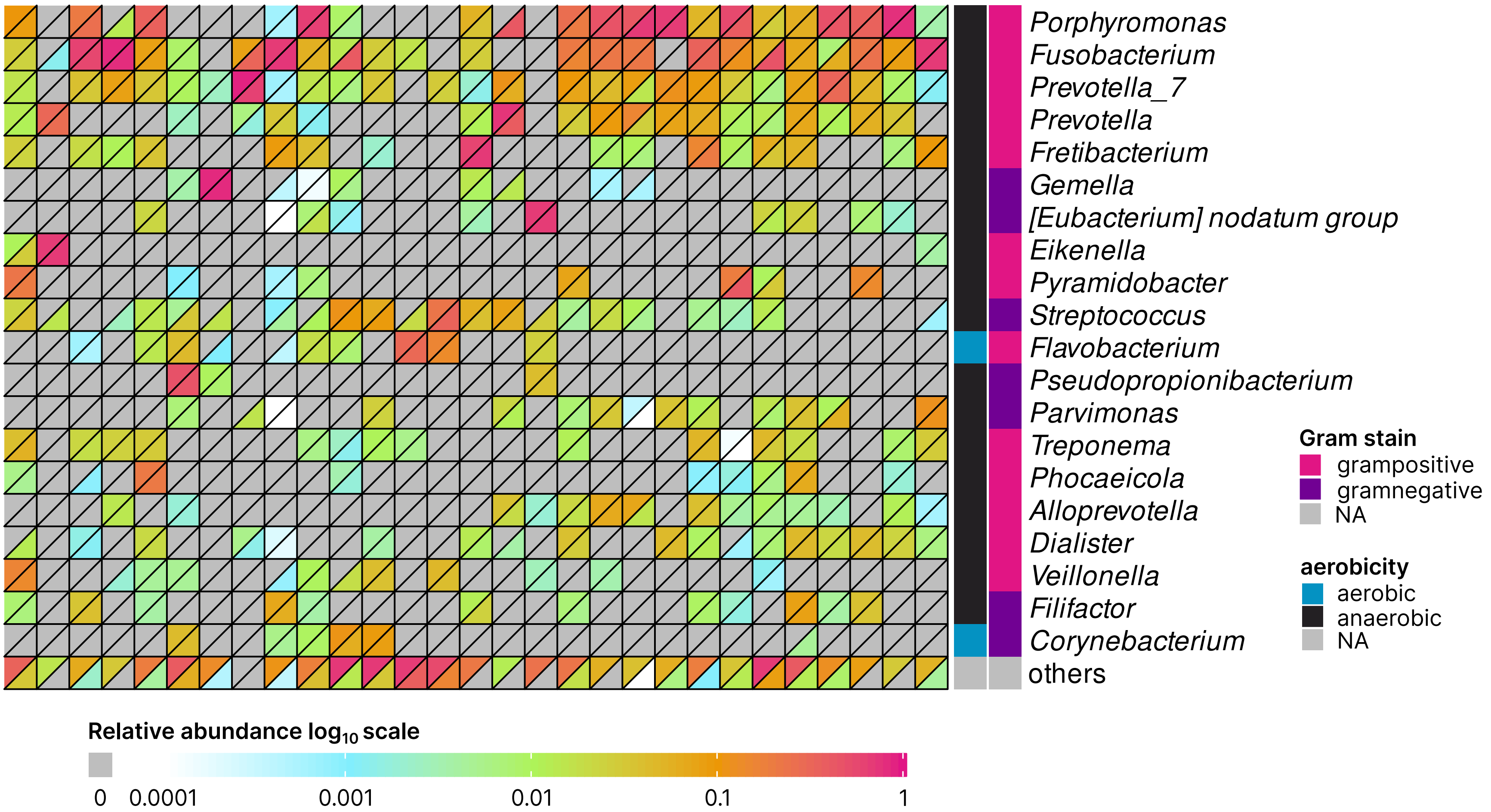

SUPPLEMENTARY FIGURE S3. Heatmap illustrating relative abundance of 25 most abundant bacterial genera (in respect to total relative abundance across all cystic fluids) in cystic fluids. The upper left triangle reflects relative abundances with the ASVs removal and the bottom right triangle “raw” data.
