# Supplementary material for: Bacterial genera in the fluids from apical periodontitis‐related radicular cysts: An observational study
Source: Int Endod J. 2025 Mar 9;58(6):902–15. doi: 10.1111/iej.14220 (PMC12065126; doi:10.1111/iej.14220)
Supplement: Supplementary file 4 — Table S1 [file IEJ-58-902-s003.pdf]

SUPPLEMENTARY TABLE S1. Primer sequences used in this study.

name	sequence
16S-F(forward)	CCTACGGGNGGCWGCAG
**name**	**Overhang + tag + spacer + sequence**
16S-F_2	TCGTCGGCAGCGTCAGATGTGTATAAGAGACAGACGAAGTCCTACGGGNGGCWGCAG
16S-F_4	TCGTCGGCAGCGTCAGATGTGTATAAGAGACAGATAATGTCCTACGGGNGGCWGCAG
16S-F_6	TCGTCGGCAGCGTCAGATGTGTATAAGAGACAGAGCCAGTCCTACGGGNGGCWGCAG
16S-F_7	TCGTCGGCAGCGTCAGATGTGTATAAGAGACAGAGTTCGTCCTACGGGNGGCWGCAG
16S-F_8	TCGTCGGCAGCGTCAGATGTGTATAAGAGACAGAATGCAGTCCTACGGGNGGCWGCAG
16S-F_9	TCGTCGGCAGCGTCAGATGTGTATAAGAGACAGAATTTAGTCCTACGGGNGGCWGCAG
16S-F_11	TCGTCGGCAGCGTCAGATGTGTATAAGAGACAGATCCTCGTCCTACGGGNGGCWGCAG
16S-F_14	TCGTCGGCAGCGTCAGATGTGTATAAGAGACAGACATTTGTCCTACGGGNGGCWGCAG
16S-F_15	TCGTCGGCAGCGTCAGATGTGTATAAGAGACAGATTGCGTGTCCTACGGGNGGCWGCAG
16S-F_16	TCGTCGGCAGCGTCAGATGTGTATAAGAGACAGATAAAGAGTCCTACGGGNGGCWGCAG
16S-F_17	TCGTCGGCAGCGTCAGATGTGTATAAGAGACAGATGCTGAGTCCTACGGGNGGCWGCAG
16S-F_18	TCGTCGGCAGCGTCAGATGTGTATAAGAGACAGACGGCTCGTCCTACGGGNGGCWGCAG
16S-F_19	TCGTCGGCAGCGTCAGATGTGTATAAGAGACAGAGATGATGTCCTACGGGNGGCWGCAG
16S-F_20	TCGTCGGCAGCGTCAGATGTGTATAAGAGACAGAATATACGTCCTACGGGNGGCWGCAG
**name**	**sequence**
16S-R	GACTACHVGGGTATCTAATCC
**name**	**Overhang + tag + spacer + sequence**
16S-R_2	GTCTCGTGGGCTCGGAGATGTGTATAAGAGACAGAGCCACCGACTACHVGGGTATCTAATCC
16S-R_4	GTCTCGTGGGCTCGGAGATGTGTATAAGAGACAGACGGCCCGACTACHVGGGTATCTAATCC
16S-R_6	GTCTCGTGGGCTCGGAGATGTGTATAAGAGACAGAAGGACCGACTACHVGGGTATCTAATCC
16S-R_7	GTCTCGTGGGCTCGGAGATGTGTATAAGAGACAGACGCTCCGACTAC

	HVGGGTATCTAATCC
16S-R_8	GTCTCGTGGGCTCGGAGATGTGTATAAGAGACAGAATTAGCCGACTACHVGGGTATCTAATCC
16S-R_9	GTCTCGTGGGCTCGGAGATGTGTATAAGAGACAGATCCTCCCGACTACHVGGGTATCTAATCC
16S-R_11	GTCTCGTGGGCTCGGAGATGTGTATAAGAGACAGATCCCTCCGACTACHVGGGTATCTAATCC
16S-R_14	GTCTCGTGGGCTCGGAGATGTGTATAAGAGACAGACCCAGCCGACTACHVGGGTATCTAATCC
16S-R_15	GTCTCGTGGGCTCGGAGATGTGTATAAGAGACAGACGGCTCCCGACTACHVGGGTATCTAATCC
16S-R_16	GTCTCGTGGGCTCGGAGATGTGTATAAGAGACAGACGACATCCGACTACHVGGGTATCTAATCC
16S-R_17	GTCTCGTGGGCTCGGAGATGTGTATAAGAGACAGATCCCGCCCGACTACHVGGGTATCTAATCC
16S-R_18	GTCTCGTGGGCTCGGAGATGTGTATAAGAGACAGAGTGCGCCCGACTACHVGGGTATCTAATCC
16S-R_19	GTCTCGTGGGCTCGGAGATGTGTATAAGAGACAGAGACTCTCCGACTACHVGGGTATCTAATCC
16S-R_20	GTCTCGTGGGCTCGGAGATGTGTATAAGAGACAGAGGGGTTCCGACTACHVGGGTATCTAATCC
